# Editorial: Telehealth innovations: improving accessibility, usability and satisfaction for older patients

**DOI:** 10.3389/fmed.2025.1774138

**Published:** 2026-01-14

**Authors:** Motti Haimi

**Affiliations:** 1 Faculty of Medicine, Technion Israel Institute of Technology, Haifa, Israel; 2 Health Systems Management, The Max Stern Yezreel Valley College, Jezreel Valley, Israel

**Keywords:** access, equity, old age, telehealth, telemedicine

The global acceleration of population aging presents unprecedented challenges for healthcare systems worldwide. In China alone, the population aged 60 and above has reached nearly 297 million, representing over 21% of the total population. Similar demographic shifts are occurring across developed nations, with projections suggesting that older adults in the United States will number 78 million by 2040. Against this backdrop, telehealth has emerged as a promising solution to extend healthcare access, support chronic disease management, and enable aging in place. Yet the promise of digital health technologies can only be realized if these innovations genuinely serve their intended users—older adults whose needs, capabilities, and contexts differ substantially from younger populations.

This Research Topic brings together five contributions that collectively illuminate the complex landscape of telehealth adoption among older adults. Spanning opinion pieces and original empirical research from diverse contexts—including the United States, China, and conflict-affected regions—these articles reveal that successful telehealth implementation requires far more than technological solutions. They demonstrate that accessibility, usability, and satisfaction are shaped by an intricate interplay of individual capabilities, psychological factors, social support systems, and broader contextual forces.

## Beyond technology: the multidimensional nature of barriers

A central theme emerging from this Research Topic is that barriers to telehealth adoption are multidimensional, encompassing physical, cognitive, technological, economic, and systemic factors. Leff et al. provide a comprehensive framework for understanding these barriers in the US context, highlighting how sensory impairments, cognitive decline, limited technology access, and gaps in digital literacy combine to create substantial obstacles. Their observation that 82% of homebound patients required assistance with telehealth visits underscores the inadequacy of technology-centric solutions that assume user independence.

The significance of digital literacy as a fundamental determinant of healthcare access is further elaborated by Zhang et al., whose cross-sectional study of over 1,100 older adults across seven Chinese cities demonstrates that digital literacy significantly influences both therapeutic and preventive healthcare utilization. Their findings reveal important heterogeneity: the impact of digital literacy is more pronounced among rural residents and those with lower economic status, suggesting that digital health interventions may inadvertently widen existing health disparities if literacy gaps are not addressed.

These barriers are amplified in crisis contexts. Haimi examines telemedicine deployment in war zones, where infrastructure disruption, displacement, and security concerns compound the challenges faced by older adults. Drawing on experiences from Ukraine and Israel, this analysis reveals how conflict strips away the support systems that older adults typically rely upon while simultaneously making remote care delivery both more necessary and more difficult.

## The psychology of adoption: trust, anxiety, and sustained engagement

Several contributions highlight that psychological factors—particularly technology anxiety and privacy concerns—play decisive roles in shaping adoption patterns and sustained use. An et al. present empirical evidence of a chain mediation pathway in which privacy concerns trigger technology anxiety, leading to dissatisfaction and ultimately discontinuous usage intention. This finding is particularly significant because it suggests that initial adoption—often the focus of implementation efforts—may be insufficient; sustaining engagement requires ongoing attention to user experience and trust.

Complementing these findings, Fu and Ma demonstrate that self-efficacy—older adults' confidence in their ability to use smart health technologies—significantly predicts behavioral intention both directly and indirectly through perceptions of ease of use. Notably, their extended UTAUT model finds that facilitating conditions (external infrastructure and support) do not significantly influence adoption intention in the Chinese context, suggesting that internal psychological resources may matter more than external provisions in shaping technology adoption decisions.

## Social support as a critical enabler

Perhaps the most consistent finding across these contributions is the pivotal role of social support in facilitating telehealth adoption. Zhang et al. demonstrate that both formal support (from healthcare institutions and government programs) and informal support (from family and friends) mediate the relationship between digital literacy and healthcare utilization, with informal support showing stronger effects. This finding resonates with Fu and Ma's observation that social influence significantly predicts adoption intention, and with Haimi's emphasis on the devastating impact of displacement and social disconnection on older adults' ability to access care in conflict settings.

Leff et al. translate these insights into practical recommendations, advocating for facilitated telehealth models that leverage community health workers, emergency medical technicians, and long-term care staff to bridge the gap between technological capability and user need. Their concept of “telehealth access hubs” in libraries and community centers offers a promising approach to democratizing access while providing the human support that many older adults require.

## Cultural context and person-centered design

The contributions from Chinese contexts reveal important cultural dimensions that shape technology adoption. Fu and Ma highlight how China's collectivist culture, with its emphasis on family support and intergenerational dependence, creates adoption pathways that differ from those observed in Western individualist societies. Similarly, Zhang et al.'s finding that technology acceptance moderates the digital literacy-healthcare relationship suggests that attitudes toward technology—shaped by cultural narratives about aging and competence—materially influence outcomes.

Multiple authors identify implicit biases, including ageism and ableism, as systemic barriers embedded within healthcare delivery and technology design. Leff et al. call attention to how provider assumptions about older adults' capabilities can lead to inappropriate exclusion from telehealth, while Haimi notes that platform designs often fail to accommodate the needs of older users. These observations underscore the need for age-friendly design principles and provider training that challenges deficit-based assumptions about older adults.

## Toward equitable telehealth: a multi-stakeholder imperative

Collectively, these contributions make clear that achieving accessible, usable, and satisfying telehealth for older adults requires coordinated action across multiple domains. Technology developers must prioritize age-friendly interfaces, simplified interactions, and robust privacy protections. Healthcare providers need training to recognize and address implicit biases while developing skills in facilitating digital engagement. Policymakers must invest in digital literacy programs, broadband access initiatives, and reimbursement structures that support facilitated telehealth models. Communities and families remain essential partners in providing the social support that enables and sustains technology use.

As we look to the future, several research priorities emerge. Longitudinal studies are needed to understand how telehealth adoption evolves over time and what factors predict sustained engagement vs. discontinuation. Implementation research should evaluate facilitated telehealth models across diverse settings and populations. And as artificial intelligence increasingly shapes digital health tools, urgent attention must be given to ensuring these technologies serve rather than further marginalize older adults.

The articles in this Research Topic ultimately remind us that telehealth innovation is not merely a technical challenge but a fundamentally human one. Success will be measured not by the sophistication of our technologies but by their ability to enhance the health, autonomy, and dignity of older adults in all their diversity.

